# Novel Foot-and-Mouth Disease Vaccine Platform: Formulations for Safe and DIVA-Compatible FMD Vaccines With Improved Potency

**DOI:** 10.3389/fvets.2020.554305

**Published:** 2020-09-25

**Authors:** John M. Hardham, Peter Krug, Juan M. Pacheco, James Thompson, Paul Dominowski, Veronique Moulin, Cyril G. Gay, Luis L. Rodriguez, Elizabeth Rieder

**Affiliations:** ^1^Zoetis Inc, Kalamazoo, MI, United States; ^2^United States Department of Agriculture (USDA) Plum Island Animal Disease Center, Agricultural Research Services, USDA, Greenport, NY, United States; ^3^United States Department of Agriculture (USDA) Office of National Programs, Agricultural Research Services, Beltsville, MD, United States

**Keywords:** FMD (foot and mouth disease), DIVA, potency, efficacy, diagnostic, vaccine platform, rapid, effective

## Abstract

Inactivated, wild-type foot-and-mouth disease virus (FMDV) vaccines are currently used to control FMD around the world. These traditional FMD vaccines are produced using large quantities of infectious, virulent, wild-type FMD viruses, with the associated risk of virus escape from manufacturing facilities or incomplete inactivation during the vaccine formulation process. While higher quality vaccines produced from wild-type FMDV are processed to reduce non-structural antigens, there is still a risk that small amounts of non-structural proteins may be present in the final product. A novel, antigenically marked FMD-LL3B3D vaccine platform under development by Zoetis, Inc. and the USDA-ARS, consists of a highly attenuated virus platform containing negative antigenic markers in the conserved non-structural proteins 3D^pol^ and 3B that render resultant vaccines fully DIVA compatible. This vaccine platform allows for the easy exchange of capsid coding sequences to create serotype-specific vaccines. Here we demonstrate the efficacy of the inactivated FMD-LL3B3D-A_24_ Cruzeiro vaccine in cattle against wild-type challenge with A_24_ Cruzerio. A proprietary adjuvant system was used to formulate the vaccines that conferred effective protection at low doses while maintaining the DIVA compatibility. In contrast to wild-type FMDV, the recombinant FMD-LL3B3D mutant viruses have been shown to induce no clinical signs of FMD and no shedding of virus in cattle or pigs when inoculated as a live virus. The FMD-LL3B3D vaccine platform, currently undergoing development in the US, provides opportunities for safer vaccine production with full DIVA compatibility in support of global FMDV control and eradication initiatives.

## Introduction

Foot and Mouth Disease Virus (FMDV) is the causative agent of a highly contagious disease that affects pigs, cattle, sheep, goats, buffalos, and other cloven-hoofed animals. The disease causes severe production losses and disrupts a wide range of agricultural, industrial, and social activities. The FMD status of a country represents the single largest barrier to trade in the agricultural sector. Estimates of the annual economic impact of FMD in endemic countries range from $6.5 to $21 billion, while the economic impact of an FMD incursion in an FMD-free country are >$1.5 billion per year (1–3).

An incursion of FMD in North America represents the single largest risk to the agricultural sector (1, 3, 4). According to the National Pork Producers Council (5), an FMD outbreak in the United States would immediately stop all export markets for U.S. pork and beef. As the export market represents ~25% of total US pork production, the outcome would be devastating to the US pork industry. Additional follow-on impacts would also result for the corn and soybean markets and have additional negative impacts to related industries (such as food processing plants, food distribution, and restaurants). The impact to the U.S. economy over 10 years is estimated to be over $128 billion for the beef and pork sectors, $44 billion for the corn sector, and $25 billion for the soybean sector with an additional loss of ~1.5 million jobs (5, 6).

Due to the risk of research on or FMD vaccine production with wild-type, virulent FMD strains, the United States has restricted the presence of the wild-type FMD viruses to only Plum Island Animal Disease Research Center in New York. This restriction has caused the United States to be reliant on overseas production of FMD vaccines. Roth and Spickler (7) stated that the United States should “seek USDA licensure of new technology FMD vaccines that could be safely manufactured in the U.S. and which are based on a platform that allows various capsid serotypes/topotypes to be inserted into the vaccine. These would then be candidates for vendor managed inventory of finished vaccine and of vaccine antigen concentrate (VAC)”.

There are currently seven immunologically distinct serotypes of FMDV that contain 60 topotypes. These serotypes, with the exception of serotype C which has not been detected in the field since 2004, circulate in seven recognized “pools” around the globe (8). The genetic and antigenic diversity of FMDV strains results in challenges with vaccine matching for effective FMD control (9). This situation leads to the necessity to maintain specific vaccines for each region. Traditional FMD vaccines (monovalent and multivalent) are comprised of virulent, wild-type viruses chemically inactivated and formulated with adjuvants. These vaccines confer protection from clinical signs of FMD caused by FMDVs closely related to the vaccine strain. However, the traditional FMD vaccines have several challenges and limitations (10–12).

There are four major drawbacks of traditional FMD vaccines that are currently commercially available. First, large quantities of infectious, virulent FMD virus are necessary to produce vaccine antigen, with the associated risk of virus escape from manufacturing facilities or incomplete inactivation during the vaccine formulation process. Therefore, traditional inactivated FMD vaccines must be manufactured in expensive biocontainment facilities utilizing virulent FMD strains. The typical volumes of culture fluids range between 1,000–5,000 liter. The associated risk of escape from the manufacturing facilities is a key reason why many countries restrict FMD vaccine production to only local endemic strains. There have been several examples of the virulent FMD viruses escaping from manufacturing facilities and causing widespread FMD outbreaks (13–16). Second, the vaccine strain must antigenically match to the wildtype FMDV responsible for the outbreak as standard vaccines may provide little or no cross-protection against different strains even within a serotype (17). High potency (emergency use) vaccines may remediate this issue somewhat (18, 19). Third, there are challenges associated with differentiating infected from vaccinated animals (DIVA) when using traditional FMD vaccines in order to serologically discern infected animals and vaccinated animals. Small amounts of residual non-structural proteins may still be present in traditional FMD vaccines, resulting in some animals with false positive results, especially if multiple revaccinations are required due to the inherent short duration of immunity of conventional vaccines (20–26). Fourth, traditional FMD vaccines may not fully protect animals from persistent infection (10, 27–32). In a 2016 study by Stenfeldt *et al*. (30), it was shown that neoteric [new or temporally acute (32)] subclinical infection or persistence resulted following challenge in similar percentage of vaccinated and non-vaccinated animals (62% in vaccinated cattle, 67% in non-vaccinated cattle), indicating that vaccination with traditional vaccines has little impact on the carrier state (30, 33, 34).

In the United States, FMDV is only one of two animal pathogens on the Select Agent List (34) requiring additional security measures. Furthermore, current U.S. law (21 U.S. Code § 113A) states that no live virus of foot-and-mouth disease may be introduced for any purpose into any part of the mainland of the United States. These U.S. regulations and restrictions create challenges for FMDV research along with the discovery, development, and manufacture of FMD vaccines.

**Figure 1:**
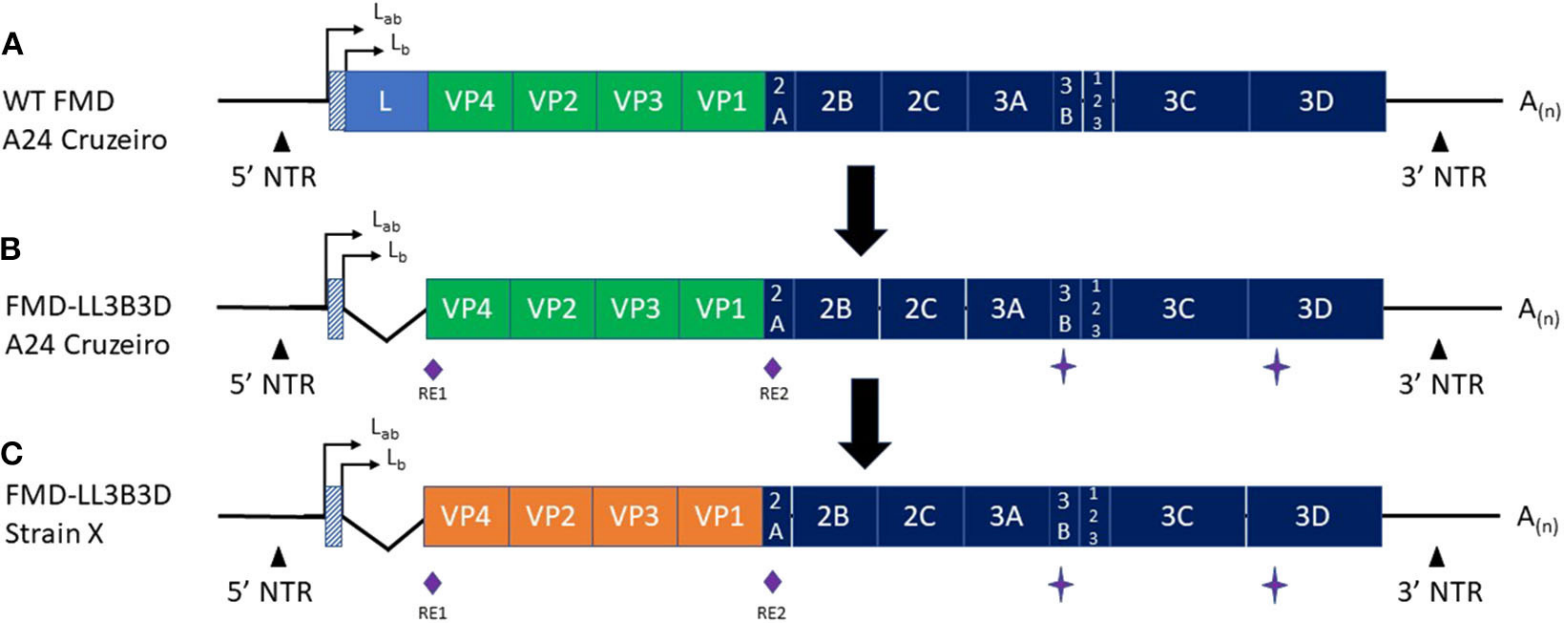
Graphic representation of the **(A)** Wild-type FMD A_24_ Cruzeiro, **(B)** FMD-LL3B3D A_24_ Cruzeiro, and **(C)** FMD-LL3B3D X genomes. The green **(A,B)** and orange **(C)** segments represent the capsid coding regions. RE1 and RE2 represent two unique restriction enzyme sites that were engineered into the genome to facilitate swapping of the capsid coding region cassettes. The star symbol represents mutations introduced into the 3B and 3D genes to negatively mark the virus rendering them fully-DIVA compatible. The angled lines **(B,C)** represent the 543-bp deletion of the leader gene rendering the resultant viruses fully attenuated. The orange region in **(C)** represents that the capsid coding region from any FMD strain may be cloned into the platform to generate a vaccine strain targeting the new strain.

For these reasons the search for alternative vaccines has been a focus of extensive research for decades. To address some of the above limitations of traditional FMD vaccines, Zoetis Inc. and the United States Department of Agriculture—Agricultural Research Services have jointly developed a safer next generation marker FMD Vaccine platform that utilizes a proprietary adjuvant system. The vaccine platform consists of an attenuated FMD A_24_ Cruzeiro virus that has been modified in three ways; (1) a 543-bp deletion of the FMDV leader sequence resulting in the complete attenuation of the FMD A24 Cruzeiro virus, (2) insertion of two unique restriction enzyme sites that flank the capsid coding region to accommodate swapping capsid coding cassettes, and (3) negative antigenic markers engineered into the non-structural proteins 3B and 3D^pol^ (35). In this study, we describe protective immune responses in cattle and DIVA capabilities after vaccination with the novel FMD-LL3B3D A_24_ Cruzeiro antigen formulated with a proprietary adjuvant. This vaccine platform allows for a rapid response capability by virtue of the easy exchange of capsid coding sequences using the unique restriction sites flanking the capsid coding region (Figure 1).

In contrast to the conventional vaccines produced with wild-type FMD viruses, the recombinant FMDV-LL3B3D platform vaccine viruses are fully attenuated as they induce no clinical signs of FMD and no shedding of virus in cattle or pigs when inoculated as a live virus (35,36, Pflaum, in preparation). As a result, this vaccine platform may use existing FMD vaccine manufacturing technology without the concerns associated with current FMD vaccine production where the risk of wild-type virus escape from the manufacturing site may cause an FMD outbreak. The finished vaccine is formulated with a proprietary adjuvant system that induces robust humoral and cellular immune responses. The FMD-LL3B3D A_24_ Cruzeiro vaccine platform strain and a large number of capsid coding cassettes were excluded from the United States Select Agent Program regulations in April 2018 (37, 38). The FMDV-LL3B3D vaccine platform is currently under development with the goal of providing a high potency, fully DIVA compatible FMD vaccines manufactured in the United States. In this manuscript, we will discuss the preliminary vaccine safety and efficacy results and assessment of the DIVA compatibility.

## Materials and Methods

### FMD-LL3B3D Vaccine Virus and Cells

The FMD-LL3B3D A_24_ Cruzeiro (also named A_24_LL3B_PVKV_3D_YR_) vaccine virus was derived in baby hamster kidney cells (BHK-21) cell monolayers as previously described (35) following virus adaptation to BHK suspension cells (sBHK). The parental construct pA24Cru cDNA infectious clone, from which the FMD-LL3B3D A_24_ Cruzeiro strain was derived, was described by Rieder *et al*. (39). Suspension BHK (sBHK) cells in Celligen BLU bioreactor vessels (Eppendorf) were expanded in serum free media (Corning) with L-Glutamine and Gentamicin (Invitrogen). Parental A_24_ Cruzeiro FMDV challenge virus (39) was obtained from the Plum Island Animal Disease Center inventory and all uses of this select agent were in compliance with regulations detailing the requirements for possession, use, and transfer for USDA select agents mandated in 9 CFR parts 331 and 121. The cell line BHK-21 was maintained in MEM (Invitrogen) medium supplemented with 10% bovine serum (GE Healthcare), 10% Tryptose Phosphate (Teknova), 2 mM L-Glutamine (Invitrogen) and 1X Antimycotic/Antibiotic (Invitrogen).

The FMD-LL3B3D vaccine platform allows derivation of other relevant strains through the use of unique restriction enzyme sites flanking the capsid coding sequence. Standard molecular biology techniques are used to exchange the capsid coding region of the FMD-LL3B3D A_24_ Cruzeiro infectious plasmid with that of other FMD strains. The resultant infectious plasmids were linearized, transcribed into RNA, and then transfected into BHK-21 cells as previously described (35).

### FMD-LL3B3D Antigen Production and Vaccine Formulation

Suspension BHK-21 cells were inoculated with the FMD-LL3B3D A_24_ Cruzeiro virus at a multiplicity of infection of 0.001 in a Celligen BLU bioreactor vessel (Eppendorf/New Brunswick) using optimal growth conditions determined previously and then virus was harvested when values of cell viability reached end points under 20%. Parameters such as pH, temperature, aeration rate and viable cell number were monitored.

The FMD-LL3B3D A_24_ Cruzeiro vaccine antigen was harvested from infected sBHK, clarified by filtering cell debris on successive capsule filters (Pall Corporation), concentrated on Hollow Fiber columns (GE Healthcare) and subjected to two chemical inactivation processes following the standard BEI inactivation protocol for vaccine preparation (40) using a 10 mM solution of 2-bromethylamine hydrobromide (BEA) in 0.7% NaOH. Virus was exposed to BEI at 25°C for up to 24 h per inactivation step. Neutralization of BEI was achieved by addition of 2% Sodium Thiosulfate (W/V). Inactivation kinetics were monitored by standard plaque assay in BHK-21 monolayers (data not shown). Complete inactivation of the bulk antigen was confirmed by a sterility assay in which three blind passages of undiluted material in BHK-21 cell monolayers were monitored for viral growth. For each passage, undiluted and diluted BEI-inactivated virus was used to infect BHK-21 monolayers followed by incubation for at least 72 h at 37°C. Vaccine antigens were stored in aliquots at −70°C until use. Specific concentrations of inactivated vaccine antigen were mixed with either Zoetis proprietary adjuvant or prepared as a water-in-oil-in-water (WOW) emulsion with Montanide ISA 206 (Seppic Paris) according to the manufacturer's instructions. The integrity of 146S particles and antigen concentration present in the formulated vaccines were determined by using 10–30% sucrose density gradients and 260 nm densitometry as previously described (35, 41, 42).

### Preliminary Vaccine Efficacy Study

All cattle experiments were performed in the BSL3Ag FMDV research facility at the Plum Island Animal Disease Center (US Department of Agriculture, Agriculture Research Services). These studies were conducted in compliance with the Animal Welfare Act (AWA), the 2011 Guide for Care and Use of Laboratory Animals, the 2002 PHS Policy for the Humane Care and Use of Laboratory Animals, and U.S. Government Principles for Utilization and Care of Vertebrate Animals Used in Testing, Research and Training (43), as well as specific animal protocols reviewed and approved by the Institutional Animal Care and Use Committee (IACUC) of the Plum Island Animal Disease Center (USDA/APHIS/AC certificate number 21-F-0001).

Holstein steers, weighing between 250 and 300 kg were identified with ear tags and housed for a week of acclimation prior to vaccination. Seven bovines in treatment groups T02 and T03 were vaccinated with the indicated dose of BEI-inactivated FMD-LL3B3D virus formulated with either ISA 206 adjuvant (T02) or Zoetis proprietary adjuvant (T03) intramuscularly (IM) in the neck. Four bovine control animals were vaccinated with sterile phosphate-buffered saline (PBS). Immediately before and at indicated times after vaccination, blood was taken for serum analysis of FMDV-specific neutralizing antibodies. On day 21 post vaccination (dpv), all cattle were challenged with homologous, wild-type FMD A_24_ Cruzeiro intradermolingually (IDL) with 10^4^ 50% bovine tongue infectious doses (BTID_50_) according to OIE guidance. The animals were monitored at 0, 4, 7, and 10 days post-challenge (dpc) for the appearance of localized and generalized lesions. Sera and temperature were collected daily. Clinical signs were scored as 1 credit for each affected foot, and presence of vesicles in the head was not considered due to lingual inoculation of challenge. FMDV RNA was measured in sera, by rRT-PCR as previously described (35, 44).

### Preliminary PD_50_ Study

To further investigate the protective immune responses to formulated FMD-LL3B3D A_24_ Cruzeiro vaccine formulated with the Zoetis proprietary adjuvant system, Holstein steers from 6 to 8 months of age were randomly assigned to one of four treatment groups (with four bovine per group): T01 received phosphate buffered saline (PBS) and served as the negative controls. Three separate vaccine dose volumes of the full dose vaccine formulated with 8 μg of hollow-fiber concentrated inactivated FMD-LL3B3D A_24_ Cruzeiro antigen and the Zoetis proprietary adjuvant system were applied intramuscularly (T02-Full dose = 2.0 ml, T03-1/4 dose = 0.5 ml, and T04-1/16 dose = 0.125 ml). Twenty-one days post-vaccination, all vaccinated and naïve animals were inoculated by the IDL route with 10^4^ BTID_50_ of homologous, wild-type FMD A_24_ Cruzeiro. All cattle were followed for 3 weeks to assess development of clinical disease as expressed by fever, nasal secretion, salivation, loss of appetite and/or lameness and to examine the presence of viral RNA in probang samples.

### Serology and Assessment of DIVA Compatibility

Serum samples from cattle in the naive (mock vaccinated) and vaccinated groups were tested for the presence of neutralizing antibodies against FMDV using a serum standard micro-neutralization test performed in 96-well plates (in quadruple replicas). End-point neutralizing titers were calculated as the reciprocal of the final serum dilution that neutralized 100 TCID_50_ of the corresponding FMDV in 50% of the wells (35, 45). The end point titer of the serum against homologous virus was calculated as the reciprocal of the last dilution of serum to neutralize 100 TCID_50_ in 50% of the wells (46). Serum samples at indicated time points were tested for the presence of antibodies against FMDV non-structural proteins (NSPs) using three commercially available competitive 3ABC Enzyme-Linked Imunosorbent Assay (cELISA) kits following the corresponding manufacturer's protocol. The three test kits were the PrioCHECK FMDV NS Antibody ELISA (Thermo Fisher Scientific) (47), VMRD FMDV Antibody Detection Kit (48), and the SERELISA FMDV NSP Antibody Competition ELISA (Zoetis Inc.). Values are cited as means ± Standard Deviations. Cellular immune responses were measured using a cell proliferation assay with PBMCs from both healthy naive and vaccinated steers (49).

### Statistical Analysis

Statistical differences of serum neutralization comparing vaccination groups was determined by 2-way ANOVA using the Tukey's multiple comparisons test in GraphPad Prism. Statistical differences of 21-day post infection non-structural protein seroconversion was determined using the unpaired *t*-test in GraphPad Prism.

## Results and Discussion

While traditional FMD vaccine formulations provide adequate safety and efficacy, there are still numerous challenges and gaps. Amongst these are use of virulent FMD strains, need for multiple doses, short duration of immunity, lack of prevention of persistence, and incomplete DIVA compatibility. In addition, timelines for development of traditional vaccines are not compatible with the need for rapid response to new or evolving FMD strains. To address these limitations, the USDA-ARS and Zoetis have developed the FMD-LL3B3D vaccine platform (Figure 1) that combines a safe and fully DIVA-compatible platform with rapid development of new inactivated FMD vaccines that are formulated with a proprietary adjuvant system which increases vaccine immunogenicity. The vaccine platform may be adapted to swap out the FMD capsid coding region while maintaining the safety and DIVA-compatibility capabilities.

Figure 1 depicts the generation of the FMD-LL3B3D vaccine platform from the wild-type FMD A_24_ Cruzeiro virus (Figures 1A,B) along with the ability to swap the capsid coding region (Figure 1C). The deletion of the leader gene renders the resultant FMD-LL3B3D vaccine viruses completely attenuated (35). Multiple FMD-LL3B3D vaccine viruses have been assessed for live virus safety in both pigs and cattle (Pflaum, in preparation). By utilizing a non-replicating, attenuated vaccine platform, events such as outbreaks associated with manufacturing facility escape (50) may be avoided. Traditional FMD manufacturers are typically limited to producing only those FMD strains that are endemic to the region. The FMD-LL3B3D vaccine platform affords the opportunity to produce global FMD vaccines from a single or a few manufacturing sites, thus improving the economics of vaccine production.

Animals that recover from natural infection are protected longer than traditionally vaccinated animals, and this is likely due to the replication of the virus in the host's cells inducing a more complete TH1 and TH2 immune response against the virus. This includes the presence of the complete repertoire of FMDV non-structural proteins, which are normally depleted in traditional vaccines or not included in recombinant FMDV vaccines. The presence of these NSPs has been shown to contain epitopes for T-cell mediated immunity, which may enhance the response to the viral infection. However, the most common assays to detect the difference between infected and vaccinated animals (DIVA) target the presence or absence of antibodies against the NSPs, making the addition of NSPs to vaccine preparation untenable. The FMD-LL3B3D vaccine platform carries mutations in the 3D polymerase (3D^pol^) and 3B non-structural proteins which act as negative markers to distinguish vaccination with this platform from natural infection, thereby making the exclusion of NSP from vaccine preparations unnecessary.

The FMD-LL3B3D vaccine platform is used to generate vaccine bulk antigen targeting a wide variety of FMD strains. The bulk antigen is blended with a proprietary vaccine adjuvant system that increases the immunogenicity of the finished product. With FMD and other antigens, the adjuvant system has been demonstrated to increase the humoral and cellular immune responses, shorten the onset of immunity, and increase the duration of immunity (unpublished data).

**Figure 2:**
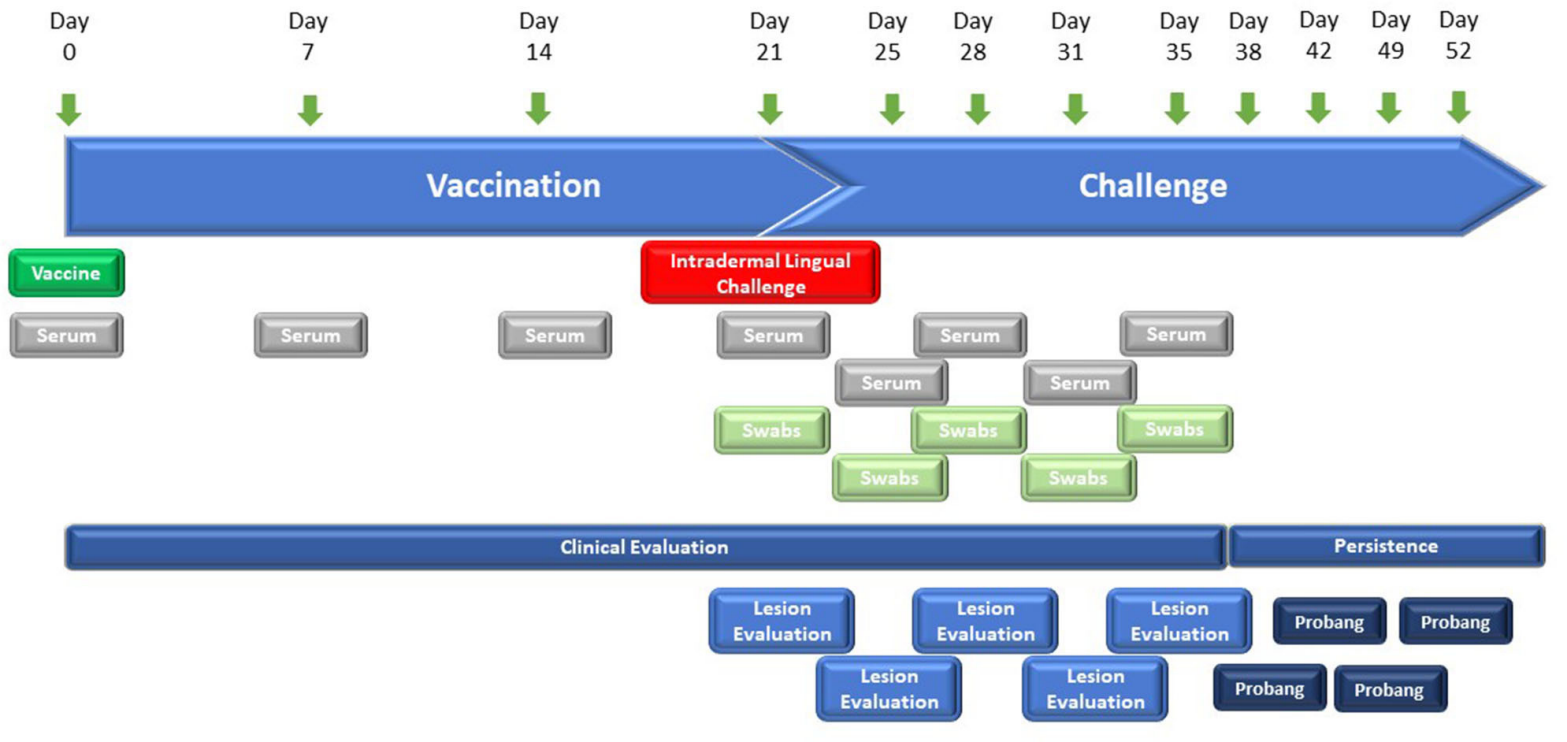
Graphic representation of the study designs for FMD-LL3B3D-based vaccine efficacy studies. Six–twelve-month-old steers were vaccinated on day 0 with the challenge occurring on day 21 where animals are intradermal-lingually challenged with 10,000 BTID_50_ of virulent, wild-type FMD virus. Serum, tonsillar swabs, and clinical observations were taken at the indicated days.

The vaccine efficacy of the FMD-LL3B3D-based vaccine formulations was demonstrated in cattle using a laboratory-based vaccine/challenge study (Figure 2). Animals were vaccinated with a single vaccine dose on day 0 and challenged via the intradermal lingual route with virulent, wild-type FMD on day 21. Clinical observations and clinical samples (serum and swabs) were taken over the next 14 days. Probang samples were taken from day 38 through day 52 to assess persistence of the challenge virus.

**Figure 3:**
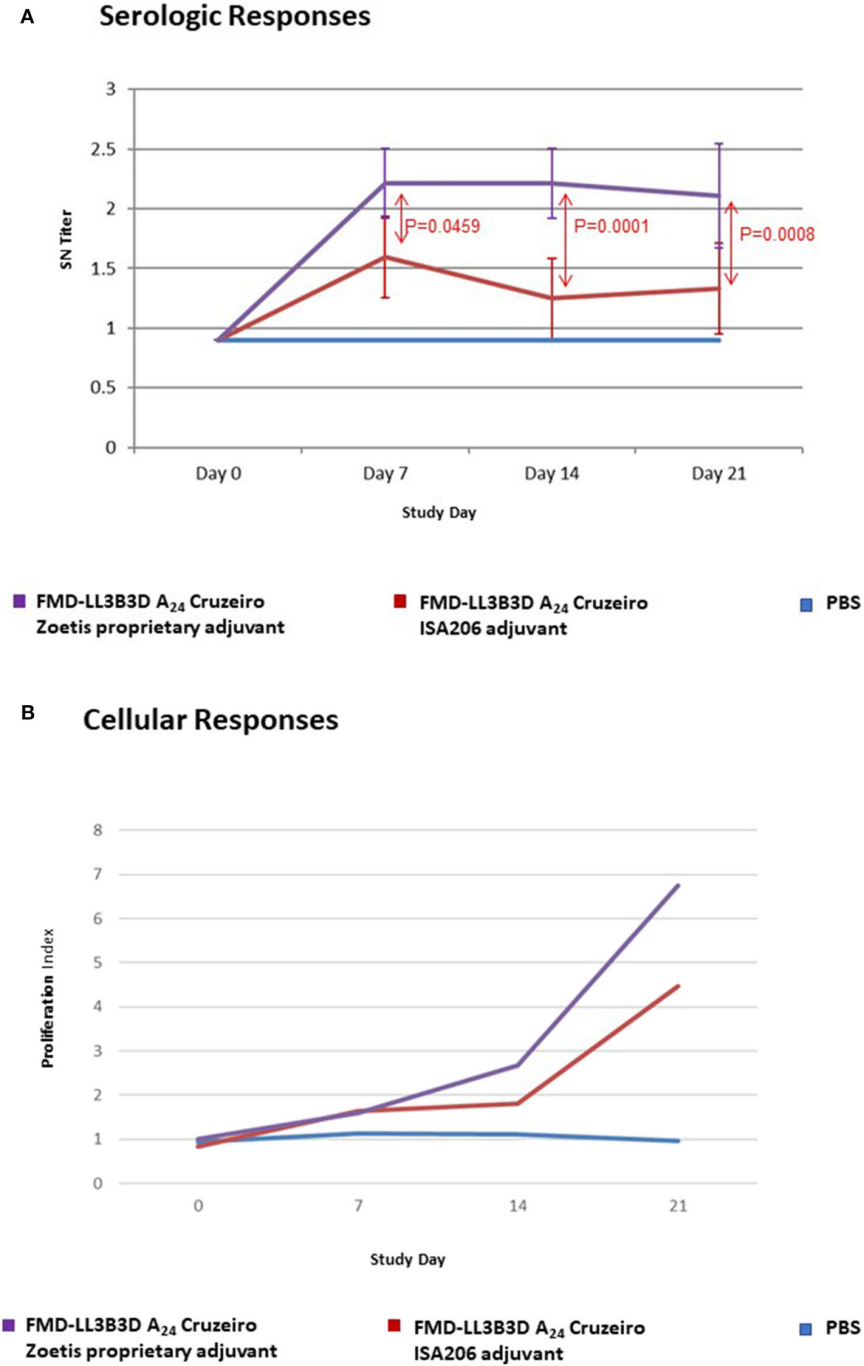
Immune responses of animals vaccinated in a preliminary vaccine efficacy study using FMD-LL3B3D A_24_ Cruzeiro antigen and a proprietary adjuvant system. **(A)** Serum neutralizing titers were measured for each vaccine group pre- and post- vaccination. FMD-LL3B3D-A_24_ vaccination induced dose-dependent levels of neutralizing antibodies. **(B)** Cellular immune responses were measured using cell proliferation assay (on PBMC from both healthy naive and vaccinated steers).

The serological and cellular immune responses to vaccination with a monovalent FMD-LL3B3D A_24_ Cruzeiro vaccine are shown on Figure 3. The serum neutralizing titer generated in response to vaccination with the FMD-LL3B3D A_24_ Cruzeiro vaccine formulated with the proprietary adjuvant system were statistically significantly higher than that generated using traditional commercial adjuvant at days 7, 14, and 21 (Figure 3A). In addition, the lymphocyte proliferation index, which represents the cellular immune response, were numerically increased in animals vaccinated using the proprietary adjuvant system vs. commercial-type adjuvant (Figure 3B).

**Figure 4:**
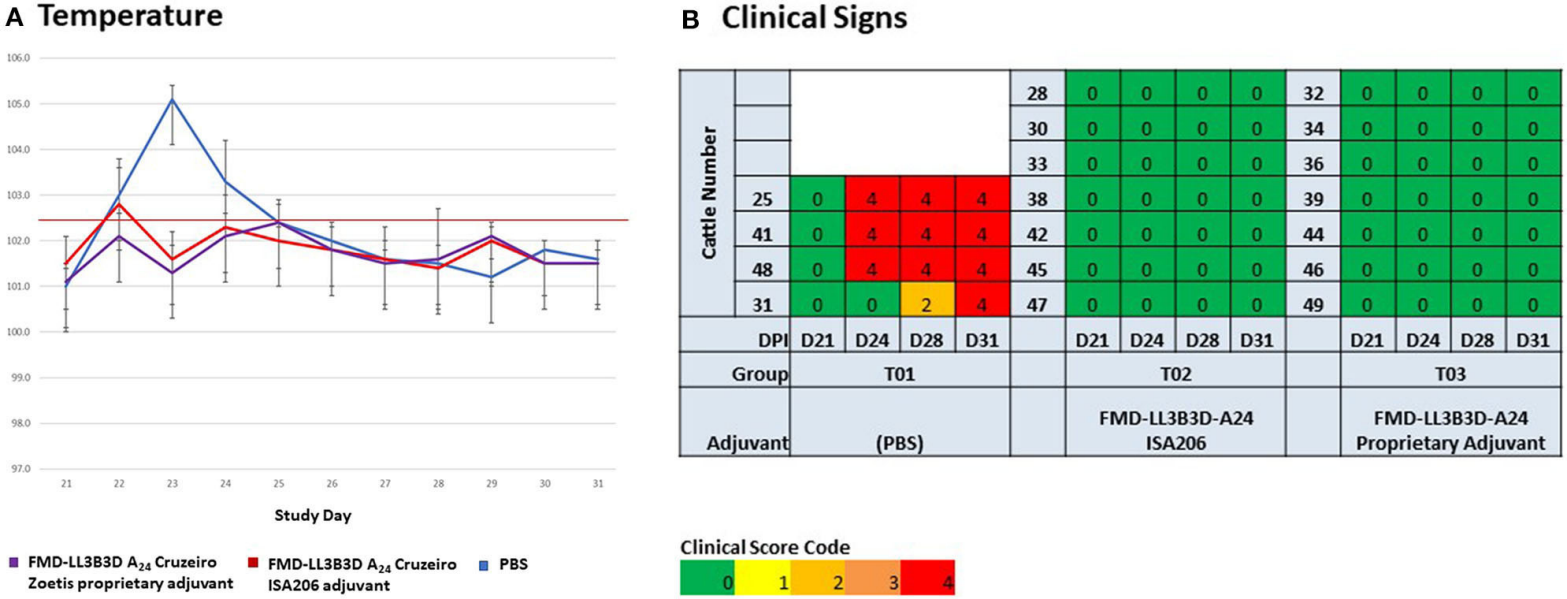
Clinical observations of animals vaccinated and challenged with virulent, wild-type FMD A_24_ Cruzeiro in an adjuvant selection study. All animals were vaccinated on day 0 and challenged on day 21. **(A)** Temperature was assessed on days 21 through 31. **(B)** Clinical observations were taken at days 21, 24, 28, and 31.

Following virulent challenge on day 21, clinical observations (temperature and clinical signs) were taken through day 31. Challenged animals that had been vaccinated with either PBS or commercial-type vaccine had elevated temperatures 1–4 days post challenge while animals vaccinated with the FMD-LL3B3D A_24_ Cruzeiro vaccine demonstrated no temperature elevations (Figure 4A). Cattle vaccinated with PBS began to show lesions consistent with FMD on their hooves and mouth starting at 3-days post challenge (Figure 4B). By day 10 post-challenge, all control animals had clinical score codes of 4. In contrast, animals vaccinated with either the commercial-type FMD A_24_ Cruzeiro vaccine or with the FMD-LL3B3D A_24_ Cruzeiro vaccine formulation did not show any lesions consistent with FMD.

**Figure 5:**
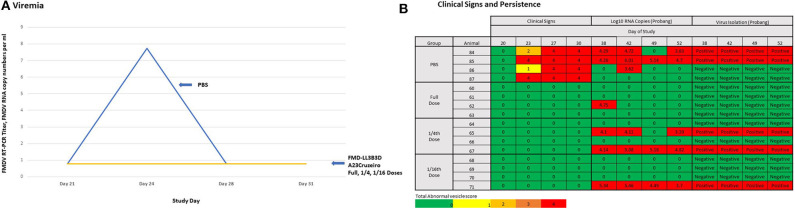
Preliminary Protective Dose 50 (PD_50_) study for the FMD-LL3B3D A_24_ Cruzeiro vaccine. Animals were vaccinated on day 0 with either a full vaccine dose of FMD-LL3B3D A_24_ Cruzeiro vaccine with a proprietary adjuvant, a quarter of a dose, or a sixteenth of a dose. All animals were then challenged on day 21. **(A)** Viremia was assessed by RT-PCR of serum samples on days 21, 24, 28, and 31. **(B)** Clinical observations were assessed on days 20, 23, 27, and 30 while challenge virus persistence was assessed by either viral isolation or RT-PCR of probang samples on days 38, 42, 49, and 52.

To determine the potency of the FMD-LL3B3D A_24_ Cruzeiro vaccine, we carried out an experiment in cattle, using full-dose, one-quarter dose, and one-sixteenth dose (2, 0.5, and 0.125 ml, respectively) of FMD-LL3B3D A_24_ Cruzeiro vaccine and challenged at 21 days post vaccination. Cattle in all three vaccinated groups challenged with virulent FMD A_24_ Cruzeiro were fully protected from both viremia (Figure 5A) and clinical signs of FMD (Figure 5B). In contrast, cattle vaccinated with PBS demonstrated a peak of viremia 3-days post challenge (Figure 5A) along with onset of lesions (Figure 5B). By day-6 post challenge, all four cattle vaccinated with PBS had clinical scores of 4 indicating that all four hooves had lesions.

Persistent infection of the challenged virus following vaccination was assessed via probang samples taken 17-, 21-, 28-, and 31-days post challenge. Probang samples were tested for the presence of FMD A_24_ Cruzeiro by RT-PCR and virus isolation. With the exception of one RT-PCR positive sample, all probang samples from cattle vaccinated with the full-dose were RT-PCR negative for FMDV and all probang samples were negative for virus isolation (Figure 5B). Two of the four cattle vaccinated with the one-quarter dose were negative by both RT-PCR and by virus isolation (Figure 5B). Three of the four cattle vaccinated with the one-sixteenth dose were negative by both RT-PCR and by virus isolation (Figure 5B). Based on these results, we can only conclude that the full-dose FMD-LL3B3D A_24_ Cruzeiro vaccine contained in excess of 16 PD_50_ per dose.

**Figure 6:**
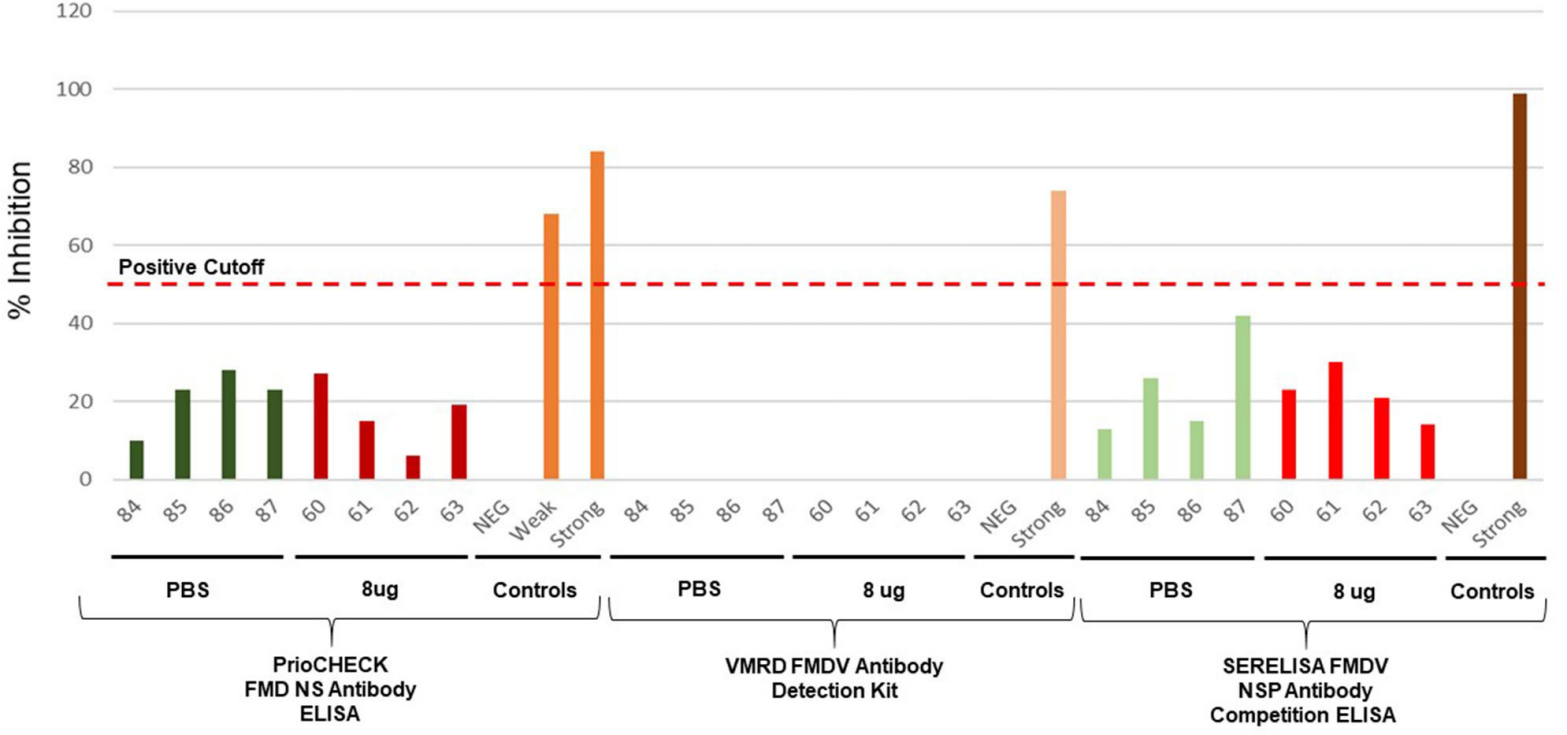
Assessment of DIVA Diagnostic compatibility of animals vaccinated with FMD-LL3B3D A_24_ Cruzeiro vaccine using the PrioCHECK FMDV NS Antibody ELISA (Thermo Fisher Scientific), Foot and Mouth Disease Virus Antibody Detection Kit cELISA (VMRD), or SERELISA® FMDV NSP Ab Competition (Zoetis).

Because traditional FMD vaccines are produced from wild-type FMDV, the bulk antigen must be purified to deplete the non-structural proteins present in the supernatant. This process is not 100% effective as there may still be some non-structural proteins present inside the intact viral particles. As such, vaccinated animals may still react to these trace non-structural proteins and present as positive results when tested by the available DIVA assays (20–26). In contrast, there is no need to deplete the non-structural proteins in the FMD-LL3B3D vaccine platform as the 3B coding region (encoding the non-structural protein) contains mutations which correspond to the epitope of the monoclonal antibodies used in the available commercial FMD DIVA diagnostic assays, thus rendering the FMD-LL3B3D vaccinated animals non-responsive (Figure 1) (35). Animals that were vaccinated with the full dose of FMD-LL3B3D A_24_ Cruzeiro vaccine were demonstrated to have reactivities similar to that of animals vaccinated with PBS in three different commercially available FMD DIVA diagnostic tests (Figure 6). This DIVA compatibility may facilitate a “vaccinate to live” policy instead of a “vaccinate to cull” as confidence in the immunologic status of the animals may be vastly increased.

**Figure 7:**
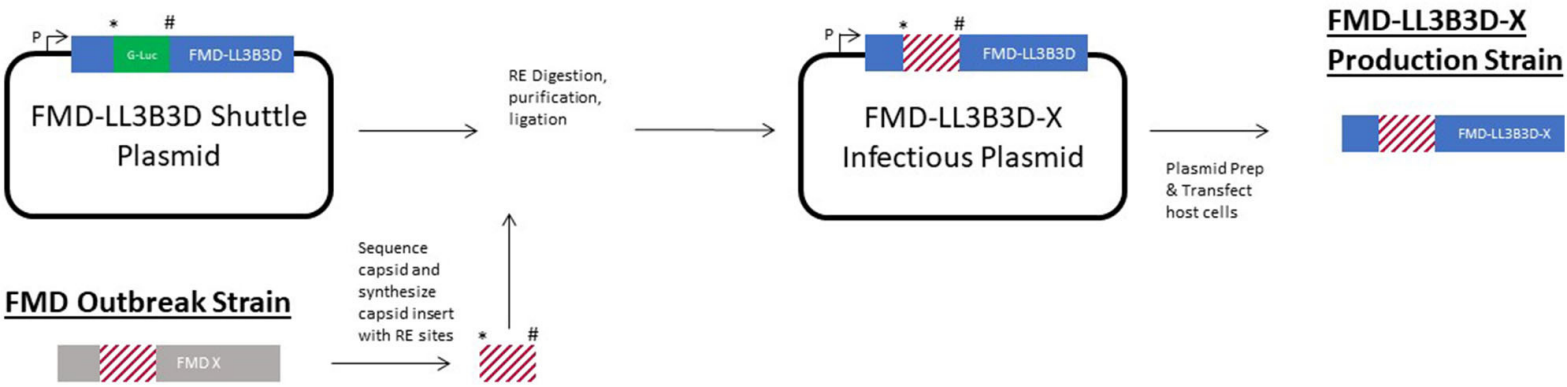
Graphic representation of the rapid response capability of the FMD-LL3B3D vaccine platform. The * and ^#^ symbols represent two unique restriction enzyme sites that were engineered into the genome to facilitate swapping of the capsid coding region cassettes.

In response to an outbreak of a novel FMD strain or incursion of a FMD strain that is not adequately covered by existing FMD vaccine banks, the FMD-LL3B3D vaccine platform is able to be utilized to rapidly generate new vaccine. A shuttle plasmid has been generated in *E. coli* in which the capsid coding region of the FMD-LL3B3D vaccine platform genome has been replace with the G-luc gene (Figure 7). This shuttle plasmid is the starting place for the rapid response capability. Upon obtaining a novel FMD strain, the capsid coding region is sequenced, and the capsid coding region is synthesized with the novel restriction sites flanking the capsid coding region. Traditional molecular biological techniques are utilized to clone the capsid coding region into the shuttle plasmid to generate a full-length plasmid construct. Following transcription, the full-length RNA is transfected into a manufacturing cell line to generate the new vaccine strain from which Premaster and Master Seeds may be derived. In this way, incursion of a new FMD strain into a FMD-free country may be initially addressed with the nearest matching vaccine and followed promptly with the specific FMD strain vaccine.

Cattle immunized with a variety of chemically inactivated FMD-LL3B3D vaccine constructs were protected from challenge with parental virus (Figures 2–5). Three commercially available FMD DIVA companion assays were shown to be compatible with the negative markers built into the FMD-LL3B3D vaccine platform and facilitate the full DIVA capability (Figure 6). Taken together, the vaccine formulations containing FMD-LL3B3D-based antigens represent an improved product profile that addresses the limitations of existing FMD vaccines and create a rapid response capability that may be utilized to promptly address incursions of new FMDV serotypes (Figure 7). This new platform technology with high potency, safe antigen production, full DIVA compatibility, and single-dose application may revolutionize the FMD vaccine market and may provide a product profile in line with National efforts to eradicate FMD.

## Data Availability Statement

The raw data supporting the conclusions of this article will be made available by the authors, without undue reservation.

## Ethics Statement

Live animals used in these studies were owned by the United States Department of Agriculture—Agricultural Research Service and the animal experiments were performed under protocols approved by the Institutional Animal Care and Use Committee of the Plum Island Animal Disease Center.

## Author Contributions

The studies were designed, directed, and coordinated by JH, ER, and LR. JH, ER, and LR provided conceptual and technical guidance for all aspects of the project. PK adapted virus to sBHK, generated preMaster seeds, determined the antigen content, and with PD formulated the vaccine. All animal experiments were conducted at the PIADC, USDA ARS BSL-3 animal facility. JP participated on the planning and conducted the animal experiment and with PK, VM, JT, ER, LR, and JH collected and analyzed the data. The manuscript was written by JH and ER with contributions and comments of all authors.

## Conflict of Interest

JH, JT, PD, and VM are employed by Zoetis Inc. The remaining authors declare that the research was conducted in the absence of any commercial or financial relationships that could be construed as a potential conflict of interest.
